# Geographical Variation and Factors Associated with Non-Small Cell Lung Cancer in Manitoba

**DOI:** 10.1155/2017/7915905

**Published:** 2017-06-21

**Authors:** David E. Dawe, Harminder Singh, Lahiru Wickramasinghe, Marshall W. Pitz, Mahmoud Torabi

**Affiliations:** ^1^Department of Hematology and Medical Oncology, CancerCare Manitoba, Winnipeg, MB, Canada; ^2^Department of Internal Medicine, Faculty of Health Sciences, University of Manitoba, Winnipeg, MB, Canada; ^3^Department of Community Health Sciences, Faculty of Health Sciences, University of Manitoba, Winnipeg, MB, Canada

## Abstract

**Background:**

Screening decreases non-small cell lung cancer (NSCLC) deaths and is recommended by the Canadian Task Force on Preventive Health Care. We investigated risk factor prevalence and NSCLC incidence at a small region level to inform resource allocation for lung cancer screening.

**Methods:**

NSCLC diagnoses were obtained from the Canadian Cancer Registry, then geocoded to 283 small geographic areas (SGAs) in Manitoba. Sociodemographic characteristics of SGAs were obtained from the 2006 Canadian Census and Canadian Community Health Survey. Geographical variation was modelled using a Bayesian spatial Poisson model.

**Results:**

NSCLC incidence in SGAs ranged from 1 to 343 cases per 100,000 population per year. The highest incidence rates were in the Southeastern, Southwestern, and Central regions of Manitoba, while most of Northern Manitoba had lower rates. Poisson regression suggested areas with higher proportions of Aboriginal people and higher average income, and immigrants had lower NSCLC incidence whereas areas with higher proportions of smokers had higher incidence.

**Conclusion:**

On an SGA level, smoking rates remain the most significant factor driving NSCLC incidence. Socioeconomic status and proportions of immigrants or Aboriginal peoples independently impact NSCLC rates. We have identified SGAs in Manitoba to target in policy and infrastructure planning for lung cancer screening.

## 1. Introduction

Lung cancer is the leading cause of cancer-related death both in Canada and worldwide [1]. In Canada, the lifetime risk of developing lung cancer is 8.4% among males and 6.9% among females [2]. The number of people diagnosed yearly with lung cancer is expected to increase by 46% between 2003–7 and 2028–32 [2]. High incidence, combined with high mortality makes lung cancer an important public health issue.

Cigarette smoking is the primary risk factor for developing lung cancer, and smoking rates have fortunately been declining over the last 30 years. However, 15–20% of adults in Canada still smoke and smoking rates have been increasing in developing nations [3, 4]. Other risk factors for lung cancer include occupational exposures (e.g., asbestos, silica, and chromium), environmental tobacco smoke, indoor coal/wood emissions, radon, family history, and pulmonary fibrosis [5, 6]. Governmental policies have aimed to decrease smoking prevalence, decrease exposure to environmental tobacco smoke, and minimize occupational exposures.

Socioeconomic status (SES) has also been described as a risk factor for lung cancer, but the definition of SES is complex and includes income, education, occupation, and employment status [7]. While these risk factors for lung cancer were initially derived using individual-level data, neighborhood level characteristics are more available for many policy planning decisions, such as infrastructure development and allocation of resources. A new infrastructure consideration related to lung cancer is the availability of computed tomography (CT) scanners that facilitate low dose CT (LDCT) screening to prevent lung cancer deaths [8–10].

Lung cancer screening with LDCT impacts mortality from non-small cell lung cancer (NSCLC), but not from small cell lung cancer [11]. The Canadian Task Force on Preventive Health Care recommends that people aged 55–74 years, who have a 30+ pack-year smoking history, and who are current smokers or quit smoking in the preceding 15 years be screened with LDCT to reduce lung cancer mortality [12]. A Health Technology Assessment by the Institute of Economics in the province of Alberta suggested that implementing an organized lung cancer screening program would increase the required number of radiologists, pathologists, and thoracic surgeons [13]. In a public payer system, these resources should be allocated where they can have the highest impact.

We used Manitoba data to investigate geographic variation in NSCLC incidence and whether prevalence of risk factors for lung cancer at a locoregional level correlated with lung cancer incidence and discuss how such data might inform resource allocation decisions related to lung cancer screening.

## 2. Methods

### 2.1. Data Sources and Study Measures

We conducted this study in the Canadian province of Manitoba. The population of Manitoba was stable across the study period ranging from 1.11 million people in 1992 to 1.2 million people in 2008 [14]. Manitoba Health provides comprehensive universal health insurance to all residents of Manitoba and maintains a population registry of permanent residents in the province. The population registry records demographic information (sex, date of birth), place of residence, migration into and out of the province, and death. We used the registry to determine the population size and distribution across the province.

Information regarding incidence of lung cancer was obtained from the Canadian Cancer Registry (CCR) housed in the Research Data Centre at the University of Manitoba. The CCR receives information regularly from all the Canadian provincial cancer registries, including the Manitoba Cancer Registry (MCR). Thus, CCR is a population-based database actively recording all cancers diagnosed in residents of Canada. Reporting of cancer cases to the individual provincial cancer registries, including MCR, is mandated by law. The coding and capture of cancer data are audited regularly by the North American Association of Central Cancer Registries. The MCR records information on all invasive cancers diagnosed among Manitobans and is 95–98% complete for cancer ascertainment [15, 16]. The MCR also receives the information on cause of death from Manitoba Vital Statistics and investigates all cases in which the reported cause of death is cancer and confirms the diagnosis.

Information regarding lung cancer was obtained from the CCR for all Manitobans diagnosed with lung cancer between 1992 and 2008. The cases of NSCLC were identified using* International Classification of Diseases for Oncology*, 3rd Edition (ICD-0-3) topography codes C340–C349 and morphology codes 8010/3, 8012/3, 8021/3, 8031/3, 8052/3, 8070–8072/3, 8074/3, 8140/3, 8250/3, 8251/3, 8260/3, 8262/3, 8310/3, 8430/3, 8480/3, 8481/3, 8490/3, and 8560/3. These codes excluded small cell lung cancer and low grade neuroendocrine (carcinoid) tumours, thereby restricting the data set to NSCLC. Age was defined as the age at diagnosis and sex was coded as male and female.

The six-digit postal code and the census subdivision code of residence at time of diagnosis were used to geocode each NSCLC case to one of 283 small geographic areas (SGAs) in Manitoba. The province of Manitoba consists of 271 census subdivisions and Winnipeg's census subdivision is further subdivided into 12 community areas. These 283 areas were the geographical units used in the analysis.

Sociodemographic characteristics (proportions of immigrants, visible minority, unemployment, higher education, Aboriginal residents, and average household annual income in each unit area of the study) were obtained from the 2006 Canadian census microdata files. Unemployment rate was defined as the percentage of the population who were unemployed and eligible for the labour force (persons actively looking for work aged 15 and older) in 2006. Proportion of Aboriginal residents was the percentage of the population reporting Aboriginal status in 2006, including North American Indian single ancestry, North American Indian and non-Aboriginal ancestries, Métis single ancestry, Métis and non-Aboriginal ancestries, Inuit single ancestry, Inuit and non-Aboriginal ancestries, and other Aboriginal multiple ancestries. Proportion of immigrants was the percentage of the population reporting in 2006 that they immigrated to Manitoba from outside Canada since 1961. Proportion of visible minority was the percentage of the population reporting visible minority status, including Chinese, South Asian, Black, Filipino, Latin American, Southeast Asian, Arab, West Asian, Korean, and Japanese. Rate of higher education was the percentage of the population 15+ with some university degree. The average annual income was average household annual income. We obtained smoking rates from the Canadian Community Health Survey (CCHS) as the percentage of population reporting daily current smoking in 2006. The CCHS is a population-based cross-sectional survey of Canadians living outside an institution and aged older than 12 years [17, 18]. The survey excludes full-time members of the Canadian Armed Forces and residents of First Nations reserves [19]. Small area smoking levels were determined by linking postal code to the proportion of respondents who reported occasional or daily current smoking in 2006.

### 2.2. Statistical Analysis

The incidence cases were standardized by age and sex to model the geographical variation of NSCLC incidence in Manitoba SGAs using a spatial Poisson model [20]. Possible unstable estimates stemming from small numbers of cases in areas with small populations were adjusted by smoothing the estimates using Bayesian spatial Poisson models. The corresponding incidence rate was smoothed by gathering information from neighboring areas in order to provide stable rate estimates [21]. The models included two random variables indicating the geographical variation and any other unspecified variation across the study region.

The Bayesian approach using Markov chain Monte Carlo (MCMC) used for this analysis [22–25] was first used by Besag et al. (BYM) [24] and the model consists of two parts. In the first part, the cases are assumed to follow a Poisson distribution with an area specific parameter *θ*_*i*_*N*_*i*_:

(1) $$\begin{array}{c} C_{i}\sim Poisson\left(\;\theta_{i}N_{i}\;\right), \end{array}$$

where *C*_*i*_ and *N*_*i*_ are the observed number of lung cancer cases and corresponding population at risk in area *i*, respectively. The second part of the model is obtained by

(2) $$\begin{array}{c} \log\left(\;\theta_{i}\;\right)=\mu +\gamma_{i}+\delta_{i}+X_{i}\beta , \end{array}$$

where *θ*_*i*_ is the relative risk (RR) in area *i*; *μ* is the overall log of mean ratio over areas; *γ*_*i*_ and *δ*_*i*_ represent geographical variation and any other unspecified variation at area *i*, respectively; *X*_*i*_ consists of risk factors (from census 2006) associated with the lung cancer outcome; and *β* is the corresponding regression coefficient. In particular, for spatial random effect *γ*_*i*_, we used the intrinsic conditionally autoregressive (ICAR) model [24] and, for the nonspatial random effect *δ*_*i*_, we used identical and independent normal distribution. The random variable *δ*_*i*_ was used in the model to capture the unspecified variation across small areas to manage possible overdispersion.

The independent normal distribution prior was assigned for fixed effects *β* with zero mean and variance 10^6^ and uniform distribution between 0 and 1000 for the standard deviations involved in the spatial and nonspatial random effects. To monitor the convergence of the model parameters, we used several diagnostic methods implemented in the Bayesian output analysis (BOA) program [26], a freely available package created for R [27]. In particular, we evaluated descriptive diagnostic tests such as the trace plots, autocorrelation of generated samples of model parameters from the posterior distribution, and convergence diagnostic tests such as Brooks, Gelman, and Rubin tests and Heidelberger and Welch test [28–30]. None of these tests indicated nonconvergence of the model parameters.

The characteristics of the SGAs evaluated in multivariable Bayesian spatial Poisson modelling included visible minority rate, proportion of residents of Aboriginal status, proportion of immigrants, unemployment rate, educational attainment, average income, and smoking rate. The NSCLC incidence cases were standardized by age and sex before incorporating into the models. The models were fitted to area-level data. The potential predictor variables (characteristics of the SGAs listed above) and the smoothed incidence rates of lung cancer were categorized based on a Jenks natural breaks classification approach [31]; the categorization of incidence rates was also used in plotting choropleth maps. ArcGIS version 10.2.2 (Environmental Systems Research Institute, USA) was used to create choropleth maps. The results of the models are presented as incidence rate ratios (IRR), which are based on the posterior probability and the corresponding 95% credible interval, which is the Bayesian equivalent to a confidence interval using the frequentist approach.

The University of Manitoba's Research Data Centre approved the study, and Statistics Canada approved data access. The models were implemented using the WinBUGS software package.

## 3. Results

**Figure 1:**
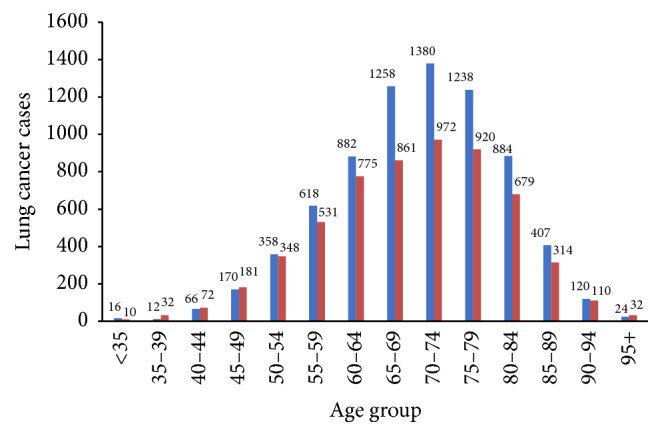
NSCLC cases in Manitoba, 1992 to 2008, by age and sex (female: red; male: blue).

Between 1992 and 2008, 12,170 NSCLC cases were diagnosed in Manitoba.  Figure 1 provides the number of cases by age and sex. The highest number of NSCLC cases occurred in the age group 70–74, and in most age groups the number of affected men was more than the affected women.  Table 1 shows NSCLC incidence rates (per 100,000) per year by age and sex and demonstrates the highest incidence rate in older men.

**Figure 2:**
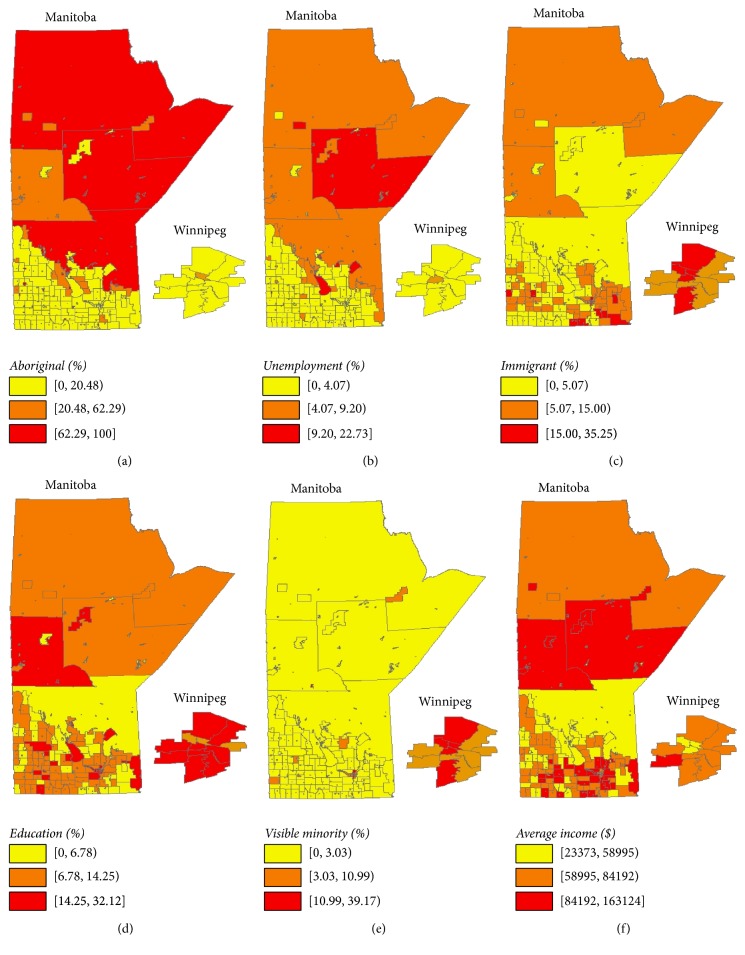
Geographical distribution of sociodemographic characteristics (shown as percentages of population in the respective regions) based on the 2006 Canadian census data: (a) Aboriginal, (b) unemployment, (c) immigrant, (d) education, (e) visible minority, and (f) average income ($).

Figure 2 depicts the sociodemographic characteristics of the SGAs in Manitoba using choropleth maps, based on 2006 Canadian Census data. The proportions of Aboriginal people and unemployment rates were highest in Northern Manitoba (Figures 2(a) and 2(b)), while the proportion of immigrants was highest in Winnipeg and Southeastern Manitoba (Figure 2(c)). As shown in Figures 2(d) and 2(f), the proportions of individuals with higher education and average income were highest in Winnipeg, Central, and Southeastern Manitoba. The proportion of visible minorities was highest in Winnipeg (Figure 2(e)).

**Figure 3:**
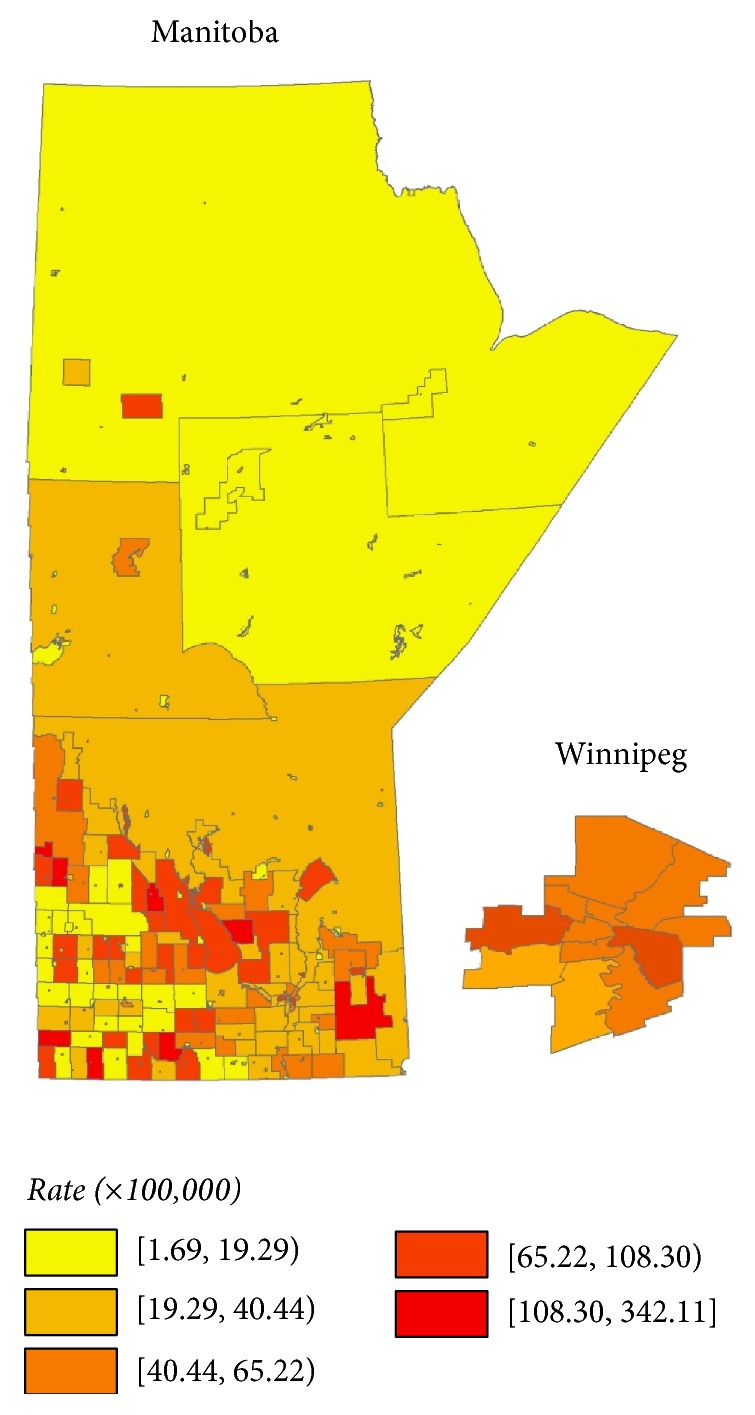
Smoothed NSCLC incidence rates (per 100,000), province of Manitoba, 1992 to 2008, age and sex standardized.

The choropleth map in Figure 3 shows the smoothed incidence rates of NSCLC across Manitoba from 1992 to 2008. The rates ranged from 1 to 343 cases (per 100,000 population) per year and were the highest in Southeastern, Southwestern, and Central regions of Manitoba and lowest in Northern Manitoba.

Table 2 represents univariate and saturated spatial Poisson regression models for age and sex standardized NSCLC incidence from 1992 to 2008. In the case of univariate models, individuals living in areas of the province with higher proportions of individuals of Aboriginal descent and areas with higher annual household income had lower rates of NSCLC incidence, whereas those residing in areas with higher proportions of active smokers had higher rates of NSCLC. Surprisingly, in univariate analysis, individuals residing in regions with higher proportions of people attaining higher education had higher rates of NSCLC; however, this effect disappeared in the saturated (multivariate) model. Visible minority, immigrant, and unemployment variables did not have a statistically significant relationship with NSCLC incidence rates in the univariate regression analysis.

The saturated Poisson regression analysis suggests that individuals residing in areas with higher proportions of Aboriginal people and higher average income and immigrants had lower NSCLC incidence rates. Individuals in areas with higher proportions of active smokers had higher rates of NSCLC. Also, most small areas in northern parts of the province had lower NSCLC incidence rates compared to those in Winnipeg. Proportions of visible minorities, unemployment rate, and educational attainment in areas of residence did not have a statistically significant relationship with NSCLC incidence rates in the saturated model. Trace plots of sociodemographic characteristics in the saturated Poisson regression model show that all model parameters converged well (supplementary appendix: Figure  A in Supplementary Material available online at https://doi.org/10.1155/2017/7915905). In stratified analysis performed for three time periods (1992–1997, 1998–2003, and 2004–2008) to investigate the time trend of NSCLS incidence rates, the results (effect size estimates) were similar in all the stratified time periods (supplementary appendix: Figures  B-D, Table  A).

## 4. Discussion

Using Bayesian spatial Poisson geographic mapping, we identified several areas within the province of Manitoba with higher incidence of NSCLC and the pattern has not changed markedly over the years. These findings were corroborated by Bayesian spatial Poisson regression analysis for predictors of NSCLC. The largest city in the province, Winnipeg, had higher rates than the remote northern part of the province. We report lower NSCLC incidence among areas with higher proportion of Aboriginal people, higher average income, lower smoking rates, and higher proportion of immigrants. Our findings are of direct relevance to developing a lung cancer screening program in the province and suggest performance of similar analyses in other jurisdictions should be able to aid the development of their screening programs.

Multiple organizations have recommended implementation of LDCT screening for lung cancer [12, 32–34]. These recommendations have led some jurisdictions to implement screening programs, but most jurisdictions have not yet implemented organized screening. In the United States, where the Center for Medicare and Medicaid Services agreed to fund screening, many people still lack insurance coverage for LDCT screening [34]. Neither the United Kingdom nor any Canadian province has put population-based screening programs in place as yet, though they are investigating feasibility. One of the concerns slowing implementation of LDCT screening has been cost effectiveness; however, Goffin et al. (2015) recently reported an expected cost of $52,000 per quality adjusted life year (QALY) in the Canadian context, which improves to $24,000/QALY when an adjunct smoking cessation program is included [35].

With more granular information, choropleth maps help end users visualize how high risk populations are distributed geographically and facilitate planning where cancer prevention and screening infrastructure can be optimally developed [36, 37]. In our study, our multivariable modelling demonstrated that smoking remains the most important factor influencing NSCLC incidence, even after adjustment for socioeconomic factors. As the primary cause of NSCLC, lowering smoking rates is a focus for lung cancer prevention [38, 39]. Since smoking cessation programs both prevent lung cancer and are important for the cost effectiveness of LDCT screening, we expect that funders are already considering this behavioral factor [32, 40]. However, we also showed that high proportions of Aboriginal peoples, high income, and a high proportion of immigrants are associated with lower NSCLC incidence. The saturated Poisson model we used allows the simultaneous integration of these multiple factors into more nuanced policy decisions. In the context of recent national recommendations to adopt LDCT screening for lung cancer, these data can help plan the distribution of screening and prevention infrastructure.

Our finding that NSCLC incidence decreases as income increases supports existing research on the impact of socioeconomic status. Sidorchuk et al. conducted a meta-analysis in 2009, identifying 64 studies and demonstrating that socioeconomic status, including income, influenced lung cancer incidence whether or not an adjustment for smoking was included [41]. A recent Canadian study demonstrated the impact of education, income, and occupation on lung cancer incidence using a large, population-based cohort with individual-level data [42]. Unfortunately, they were unable to adjust for smoking history. Our focus on Manitoba provides a smaller sample size than the Canadian study, but income remained a predictor after adjusting for SGA smoking rates.

Immigrant populations in economically developed nations appear to be at lower risk of cancer overall but at higher risk for cancers caused by microbial infections, such as liver and cervical cancers [43, 44]. In Canada, an inverse correlation of cancer incidence rates and the area concentration rate of foreign-born residents has been demonstrated. This trend was evident for lung cancer Canada-wide, regionally, and now in our study [45]. A recent study shows higher lung cancer mortality in native-born versus foreign-born Americans and attributes 50–75% of the difference in overall life expectancy among foreign-born Americans to smoking [46]. Similarly, Aboriginal/Indigenous peoples typically have lower rates of lung cancer than settler populations [47–51]. The risk of developing lung cancer does appear to be equalizing over time, with some jurisdictions reporting higher lung cancer incidence rates among Aboriginal peoples. Changing cigarette smoking rates likely drive these trends [48–51].

Governmental analyses of lung cancer incidence and risk factors in Manitoba and other provinces have focused primarily on large Regional Health Authorities (RHAs) [52–54]. There are only five RHAs in the province of Manitoba and their large size limits the utility of such an examination. For example, a previous report suggested that Northern Manitoba had the highest incidence of lung cancer, whereas our analysis indicates that most SGAs in the North have low incidence of NSCLC [52]. This suggests that the small SGAs with very high incidence and/or lack of adjustment for random effects in the statistical models could drive the results for sparsely populated areas. Using much smaller geographic areas and more robust methodology (e.g., Bayesian spatial Poisson models) allows for more accurate assessment of variation and risk and should be of greater utility in evaluation and policy planning. In British Columbia, groups have used geographic mapping analyses to propose optimal sites of radiation therapy centers and palliative care services [55, 56]. These groups used analyses based on travel time between patient residence and a new center. While this analysis differs from ours in detail, the principles of providing a visual representation of priority areas, incorporating small area characteristics, and using geographic mapping techniques are shared. We believe public health efforts to reduce the morbidity and mortality of NSCLC should focus on both the areas with high incidence of NSCLC (to reduce mortality due to NSCLC) and those with risk factors, for example, high smoking rates (to reduce the development and hence morbidity due to NSCLC). We suggest that geographic mapping techniques should be routinely used in health infrastructure planning.

The strengths of our study include the high reliability of NSCLC case acquisition through the MCR/CCR and the linkage of these data to postal code, census subdivision, and 2006 Census microdata. These are all high quality data sets that minimize errors in categorization and geographic location and ensure that almost no NSCLC cases have been missed. Further, inclusion of smoking status from the Canadian Community Health Survey allowed us to adjust for lung cancer's most important risk factor in our saturated model. Limitations of our study include that we were not able to adjust for some important lung cancer risk factors, such as radon and occupational exposures. Interestingly, some of the SGAs with high NSCLC incidence may correlate with previous reports of areas with high radon levels [57]. The acquisition of data on radon exposure in the future would facilitate a more complete analysis. The retrospective nature of the study meant that we were relegated to assessing only variables included in the linked databases. For example, the CCHS did not include smoking data for First Nations people on reserves but does include data for off-reserve First Nations people. While this limits the applicability of our results to these populations, the high rates of smoking for First Nations people already mandate attention for lung cancer prevention [58–60]. Our analyses were performed at small area level; by design we were interested in the characteristics of the area of residence, but this means that we did not have individual-level data. Individual data would have helped develop more robust models for evaluating the association between lung cancer incidence and risk factors; however, individual-level risk factor data is usually not available on a population basis. Risk factor data at the SGA level is available to policy planners, can be geographically mapped, still correlates with NSCLC incidence, and facilitates infrastructure planning as we have detailed above.

## 5. Conclusion

Our study demonstrates that, even at a small area of residence level, smoking rates remain the most significant factor driving NSCLC incidence. Regional socioeconomic status, proportion of immigrants, and proportion of Aboriginal peoples impact NSCLC incidence in Manitoba. Our methods can identify small areas with higher NSCLC rates, which should help better target policy and infrastructure planning with regard to lung cancer screening or prevention strategies. We propose that these methods should be more widely used to aid the programs developing in other jurisdictions.

## Supplementary Material

Trace plots of sociodemographic factors, additional choropleth maps, and regression analysis.

## Figures and Tables

**Table 1:** NSCLC counts, incidence rates (per 100,000), and population numbers in Manitoba (1992–2008) by age and sex.

Age at diagnosis	Male			Female		
	Count	Rate	Population	Count	Rate	Population
<35	1	0.348	287436	1	0.359	278686
35–39	1	2.265	44145	2	4.548	43980
40–44	4	9.031	44293	4	9.049	44206
45–49	10	24.362	41048	11	26.928	40849
50–54	21	60.327	34810	20	57.256	34931
55–59	36	125.589	28665	31	106.838	29016
60–64	52	219.724	23666	46	188.124	24452
65–69	74	366.210	20207	51	229.678	22205
70–74	81	467.344	17332	57	270.810	21048
75+	157	546.562	28725	121	256.519	47170
Total	437	76.623	570327	344	58.649	586543

**Table 2:** Univariate and saturated Poisson regression analyses of age and sex standardized NSCLC incidence rate ratios, 1992 to 2008, in Manitoba.

	Univariate IRR (CI)		Saturated IRR (CI)	
Visible minority, %^1^				
<3.03	1.00 (−)		1.00 (−)	
3.03 to <10.99	1.304	(0.992, 1.662)	0.898	(0.501, 1.509)
10.99 to 39.17	1.253	(0.916, 1.686)	0.804	(0.406, 1.454)
Aboriginal status, %				
<20.48	1.00 (−)		1.00 (−)	
20.48 to <62.29	1.280	(0.900, 1.761)	1.160	(0.764, 1.643)
62.29 to 100	**0.218**	** (0.077, 0.430)**	**0.225**	** (0.063**, **0.520)**
Immigrant, %				
<5.07	1.00 (−)		1.00 (−)	
5.07 to <15.00	1.059	(0.793, 1.431)	0.736	(0.492, 1.019)
15.00 to 35.25	0.981	(0.684, 1.346)	**0.497**	** (0.303**, **0.762)**
Unemployment rate, %				
<4.07	1.00 (−)		1.00 (−)	
4.07 to <9.20	0.881	(0.600, 1.224)	0.968	(0.616, 1.445)
9.20 to 22.73	0.550	(0.130, 1.303)	2.273	(0.522,6.606)
Education, %				
<6.78	1.00 (−)		1.00 (−)	
6.78 to <14.25	1.357	(0.939, 1.959)	0.862	(0.589, 1.235)
14.25 to 32.12	**1.531**	** (1.082**, **2.152)**	1.068	(0.602, 1.722)
Annual income, $				
23,373 to <58.995	1.00 (−)		1.00 (−)	
58.995 to <84.192	**0.796**	** (0.634, 0.985)**	**0.638**	** (0.435**, **0.909)**
≥84,192	**0.499**	** (0.356, 0.688)**	**0.450**	** (0.269**, **0.700)**
RHA				
Winnipeg	1.00 (−)		1.00 (−)	
North	0.344	(0.262, 1.210)	**0.497**	** (0.324**, **0.621)**
South	0.622	(0.374, 1.802)	0.647	(0.364, 1.696)
East	0.903	(0.373, 1.799)	0.850	(0.149, 1.837)
West	1.004	(0.364, 1.756)	0.747	(0.289, 1.860)
Smoking, %				
0	1.00 (−)		1.00 (−)	
0 to < 6.30	**3.531**	** (1.917, 6.152)**	**3.435**	** (1.899**, **5.950)**
6.30 to 53.48	**5.500**	** (3.098, 9.410)**	**5.050**	** (2.914**, **8.952)**

^1^All percentages refer to the proportion of the population with the characteristic in the area of residence; IRR: incidence rate ratio; CI: confidence interval.
