# Supplementary material for: Geographical Variation and Factors Associated with Non-Small Cell Lung Cancer in Manitoba
Source: Can Respir J. 2017 Jun 21;2017:7915905. doi: 10.1155/2017/7915905 (PMC5499243; doi:10.1155/2017/7915905)
Supplement: Supplementary file 1 — Trace plots of sociodemographic factors, additional choropleth maps, and regression analysis. [file 7915905.f1.docx]

**Supplementary Appendix:**

**Figure A:** Trace plots of sociodemographic characteristics in the saturated Poisson regression analysis of age and sex standardized NSCLC incidence rates, 1992-2008, in Manitoba

Visible Minority

Smoking

Immigrant

Regional Health Authority

Average Income

Unemployment

Education

Aboriginal

**Figure B:** Smoothed NSCLC incidence rates (per 100,000), province of Manitoba, 1992 to 1997, age-and sex-adjusted.

The choropleth map in Supplementary Figure 1 shows the smoothed incidence rates of NSCLC across Manitoba from 1992 to 1997. The rates ranged from 2 to 251 (per 100,000) per year and the highest rates were in the south-western and central parts of Manitoba, while some of northern part of Manitoba had lower rates of NSCLC.

**
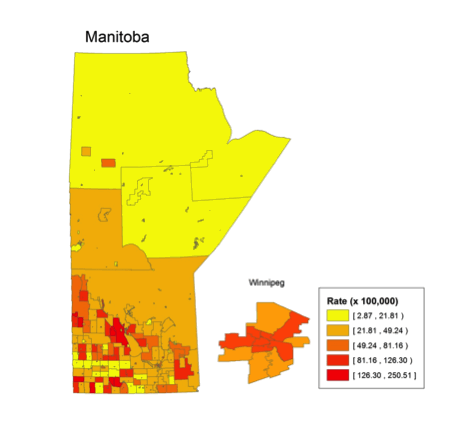
**

**Figure C:** Smoothed NSCLC incidence rates (per 100,000), province of Manitoba, 1998 to 2003, age- and sex-standardized .

The choropleth map in Supplementary Figure 2 shows the smoothed incidence rates of lung cancer across Manitoba from 1998 to 2003. The rates ranged from 5 to 212 (per 100,000) per year and the highest rates were in the south-western, south-western and central parts of Manitoba, while some of northern part of Manitoba had lower rates of NSCLC.


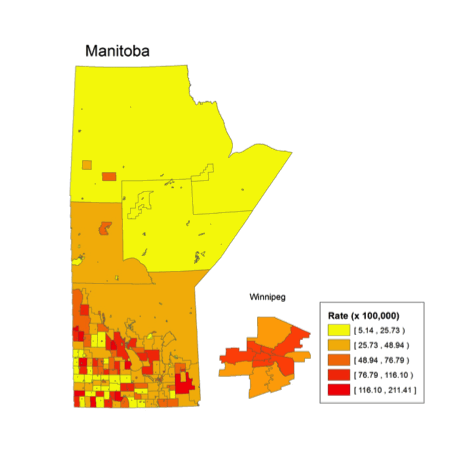


**Figure D:** Smoothed NSCLC incidence rates (per 100,000), province of Manitoba, 2004 to 2008, age- and sex-standardized .

The choropleth map in Supplementary Figure 3 shows the smoothed incidence rates of NSCLC across Manitoba from 2004 to 2008. The rates ranged from 3 to 140 (per 100,000) per year and the highest rates were in the south-eastern and central parts of Manitoba, while some of northern part of Manitoba had lower rates of NSCLC.


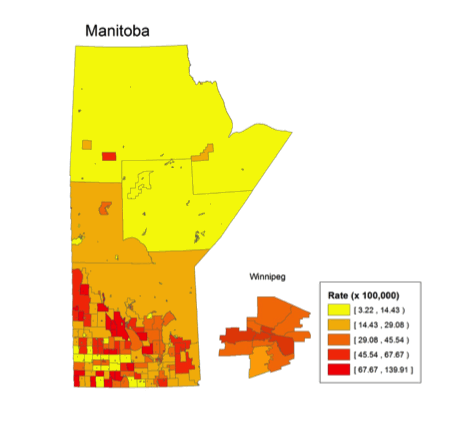


Table A represents the *saturated* (age- and sex- standardized ) Poisson regression models for NSCLC cases for the three different time periods: 1992 to 1997, 1998 to 2003, and 2004 to 2008. For the time period 1992-1997, areas with higher proportions of visible minorities, immigrants, aboriginals, and average income had lower rates of NSCLC incidence whereas areas with higher proportions of smoking had high rates of NSCLC incidence. Also, northern part of the province had lower rates of NSCLC incidence compared to Winnipeg. Unemployment rate and education did not have a relationship with NSCLC incidence rates in the saturated model.

| **Table A:** | |  | | | | |  |  | |  | |  |  |  | |  | |  |
| --- | --- | --- | --- | --- | --- | --- | --- | --- | --- | --- | --- | --- | --- | --- | --- | --- | --- | --- |
|  |  |  |  | | Age-sex-standardized *saturated* Poisson regression analysis of NSCLC cases, three time periods 1992-1997, 1998-2003, and 2004-2008, in Manitoba | | | | | | | | | | | | | |
|  | | | | **1992-1997**  **IRR(CI)** | | | | | | | **1998-2003**  **IRR(CI)** | |  |  | **2004-2008**  **IRR(CI)** | |  | |
| **Visible minority, %** | | | |  | |  | | |  | |  | |  |  |  | |  | |
| <3.03 | | | | 1.00 (-) | |  | | | 1.00(-) | |  | | 1.00(-) |  |  | |  | |
| 3.03 to <10.99 | | | | 0.672 | | (0.356, 1.100) | | | 0.731 | | (0.436,1.138) | | 0.920 |  | (0.489,1.589) | |  | |
| 10.99 to 39.17 | | | | 0.554 | | (0.264, 0.973) | | | 0.687 | | (0.376.1.180) | | 0.861 |  | (0.414,1.597) | |  | |
| **Aboriginal Status, %** | | | |  | |  | | |  | |  | |  |  |  | |  | |
| <20.48 | | | | 1.00 (-) | |  | | | 1.00(-) | |  | | 1.00(-) |  |  | |  | |
| 20.48 to <62.29 | | | | 1.214 | | (0.861, 1.670) | | | 1.228 | | (0.854,1.694) | | 1.093 |  | (0.729,1.587) | |  | |
| 62.29 to 100 | | | | 0.226 | | (0.087, 0.562) | | | 0.471 | | (0.177,0.954) | | 0.414 |  | (0.128,1.015) | |  | |
| **Immigrant, %** | | | |  | |  | | |  | |  | |  |  |  | |  | |
| <5.07 | | | | 1.00 (-) | |  | | | 1.00(-) | |  | | 1.00(-) |  |  | |  | |
| 5.07 to <15.00 | | | | 0.741 | | (0.512, 1.204) | | | 0.856 | | (0.625,1.311) | | 0.805 |  | (0.528,1.141) | |  | |
| 15.00 to 35.25 | | | | 0.534 | | (0.342, 0.804) | | | 0.560 | | (0.370,0.813) | | 0.599 |  | (0.363,0.939) | |  | |
| **Unemployment rate, %** | | | |  | |  | | |  | |  | |  |  |  | |  | |
| <4.07 | | | | 1.00 (-) | |  | | | 1.00(-) | |  | | 1.00(-) |  |  | |  | |
| 4.07 to <9.20 | | | | 1.099 | | (0.728, 1.579) | | | 0.899 | | (0.601,1.311) | | 1.269 |  | (0.786,1.982) | |  | |
| 9.20 to 22.73 | | | | 1.270 | | (0.250, 3.320) | | | 1.153 | | (0.268,2.869) | | 1.663 |  | (0.234,4.894) | |  | |
| **Education, %** | | | |  | |  | | |  | |  | |  |  |  | |  | |
| <6.78 | | | | 1.00 (-) | |  | | | 1.00(-) | |  | | 1.00(-) |  |  | |  | |
| 6.78 to <14.25 | | | | 0.939 | | (0.642, 1.358) | | | 0.866 | | (0.599,1.269) | | 1.089 |  | (0.686,1.618) | |  | |
| 14.25 to 32.12 | | | | 1.136 | | (0.654, 1.875) | | | 1.129 | | (0.643,1.816) | | 1.141 |  | (0.599,2.027) | |  | |
| **Annual income, $** | | | |  | |  | | |  | |  | |  |  |  | |  | |
| 23,373 to <58.995 | | | | 1.00 (-) | |  | | | 1.00(-) | |  | | 1.00(-) |  |  | |  | |
| 58.995 to <84.192 | | | | 0.614 | | (0.428, 0.854) | | | 0.644 | | (0.452,0.892) | | 0.922 |  | (0.598,1.313) | |  | |
| >=84,192 | | | | 0.416 | | (0.254, 0.642) | | | 0.436 | | (0.270,0.689) | | 0.688 |  | (0.389,1.073) | |  | |
| **RHA** | | | |  | |  | | |  | |  | |  |  |  | |  | |
| Winnipeg | | | | 1.00 (-) | |  | | | 1.00(-) | |  | | 1.00(-) |  |  | |  | |
| North | | | | 0.448 | | (0.313, 0.591) | | | 0.591 | | (0.325,1.270) | | 0.563 |  | (0.322,0.956) | |  | |
| South | | | | 0.456 | | (0.106,1.151) | | | 0.638 | | (0.008,1.270) | | 0.577 |  | (0.013,1.112) | |  | |
| East | | | | 0.563 | | (0.230,1.622) | | | 0.787 | | (0.009,1.546) | | 0.857 |  | (0.044,1.579) | |  | |
| West | | | | 0.701 | | (0.489,2.397) | | | 0.800 | | (0.012,1.545) | | 0.891 |  | (0.005,1.753) | |  | |
| **Smoking, %** | | | |  | |  | | |  | |  | |  |  |  | |  |  |
| 0 | | | | 1.00 (-) | |  | | | 1.00(-) | |  | | 1.00(-) |  |  | |  |  |
| 0 to < 6.30 | | | | 2.863 | | (1.735, 4.589) | | | 2.701 | | (1.573,4.506) | | 3.142 |  | (1.540,5.853) | |  |  |
| 6.30 to 53.48 | | | | 4.112 | | (2.491, 6.628) | | | 4.140 | | (2.413,6.990) | | 4.652 |  | (2.397,8.258) | |  |  |

IRR = incidence rate ratio, CI = confidence interval

For the time period 1998-2003, areas with higher proportions of immigrants, aboriginals, and average income had lower rates of NSCLC incidence whereas the areas with higher proportions of smoking had high rates of NSCLC incidence. Visible Minorities, unemployment rate, RHA and education did not have a relationship with NSCLC incidence rates in the saturated model.

For the time period 2004-2008, areas with higher proportions of immigrants had lower rates of NSCLC incidence whereas areas with higher proportions of smoking had high rates of NSCLC incidence. Also, northern part of the province had lower rates of NSCLC incidence compared to Winnipeg. Visible minorities, unemployment rate, aboriginals, average income and education did not have a relationship with NSCLC incidence rates in the saturated model.
